# Dystonic storm: a practical clinical and video review

**DOI:** 10.1186/s40734-017-0057-z

**Published:** 2017-04-28

**Authors:** Pichet Termsarasab, Steven J. Frucht

**Affiliations:** Movement Disorder Division, Department of Neurology, Icahn School of Medicine at Mount Sinai, 5 East 98th St, New York, NY 10029 USA

**Keywords:** Dystonic storm, Status dystonicus, Dystonia, Treatment, Early recognition, Deep brain stimulation

## Abstract

Dystonic storm is a frightening hyperkinetic movement disorder emergency. Marked, rapid exacerbation of dystonia requires prompt intervention and admission to the intensive care unit. Clinical features of dystonic storm include fever, tachycardia, tachypnea, hypertension, sweating and autonomic instability, often progressing to bulbar dysfunction with dysarthria, dysphagia and respiratory failure. It is critical to recognize early and differentiate dystonic storm from other hyperkinetic movement disorder emergencies. Dystonic storm usually occurs in patients with known dystonia, such as DYT1 dystonia, Wilson’s disease and dystonic cerebral palsy. Triggers such as infection or medication adjustment are present in about one-third of all events. Due to the significant morbidity and mortality of this disorder, we propose a management algorithm that divides decision making into two periods: the first 24 h, and the next 2–4 weeks. During the first 24 h, supportive therapy should be initiated, and appropriate patients should be identified early as candidates for pallidal deep brain stimulation or intrathecal baclofen. Management in the next 2–4 weeks aims at symptomatic dystonia control and supportive therapies.

**Electronic supplementary material:**

The online version of this article (doi:10.1186/s40734-017-0057-z) contains supplementary material, which is available to authorized users.

## Introduction

Dystonic storm is a malignant hyperkinetic movement disorder emergency in which rapid deterioration of dystonia requires emergent intervention [1]. Jankovic and Penn first described the disorder in 1982 in an 8-year-old boy with dystonia musculorum deformans who developed rapid marked worsening of dystonia, elevated serum creatine kinase (CK) and myoglobinuria [2]. This entity was later referred to as “desperate dystonia” [3] or “dystonic storm” [4]. In 1998, Manji described a series of 12 patients with “status dystonicus”, and their definition has been referenced in subsequent reports [5–7].

In this review, we summarize the clinical features of dystonic storm, review its differential diagnosis and pathophysiology, and present a practical guideline for the treating clinician, aimed at identifying patients who are surgical candidates and managing those who are not with medication and supportive care.

## Review

### Recognition of dystonic storm

In addition to severe generalized dystonia, symptoms and signs of dystonic storm include fever, tachycardia, tachypnea or respiratory change, hypertension, sweating and autonomic instability. Dystonia can be tonic (i.e. sustained posturing) or phasic (i.e. irregular jerking). The largest study of 89 episodes of storm revealed that most were tonic (69%), and phasic storms occurred more commonly in females and patients with secondary dystonia [6]. Other movement disorders such as chorea, ballism or myoclonus may accompany dystonia. Bulbar impairment such as dysarthria, dysphagia and respiratory failure are common, and these require close monitoring in the intensive care unit. Pain is also common, and often requires aggressive symptomatic control. Laboratory findings include leukocytosis, elevated serum CK, high C-reactive protein, myoglobinemia, and myoglobinuria that may be severe enough to lead to renal failure and acidemia.

## Differential diagnosis of dystonic storm

Several conditions may mimic dystonic storm, presenting with hyperthermia, elevated serum CK, and/or muscle rigidity. It is important to differentiate dystonic storm from other hyperkinetic movement disorder emergencies [8] (Table 1 and Additional file 1: Video segment 1). The phenomenology of the underlying movement disorder, associated neurological symptoms and signs, age of the patient, history of triggers, and time course are helpful clues. Compared to other movement disorder emergencies, dystonic storm typically occurs in the pediatric age group. Triggers may or may not be present (see below), and storm usually develops quickly over hours to several days.

**Table 1:** Differential diagnosis of dystonic storm

Entity	Age	Trigger	Time course	Movement disorder phenomenology	Other neuro signs	Altered mental status	Autonomic instability
Dystonic storm	P	+/−	Hours-Days	Dystonia +/− chorea	−	−	+
Choreic storm	P-YA	+/−	Hours-Days	Chorea	−	−	−
Oculogyric crisis	All	+++	Acute	Dystonia	Oculogyria	−	+/−
Neuroleptic malignant syndrome	A	+++	Days-Weeks	Parkinsonism	−	+	+
Serotonin syndrome	All	+++	Hours-Days	Myoclonus	−	+	+
Lethal catatonia	All	+/−	Hours-Days	Parkinsonism	−	+	+
Malignant hyperthermia	All	+++	Acute	−	−	−	−
Drug intoxication	All	+++	Acute	−	Psychosis	+	+/−
Intrathecal baclofen withdrawal	P-YA	+++	Acute-Hours	−	−	+	+
Delirium tremens	A	+++	Acute	Myoclonus	Psychosis	+	+
Autoimmune encephalitis (e.g. anti-NMDA)	P-YA	−	Days-Weeks	Chorea	Psychosis	+	+

This table provides the list of differential diagnosis of dystonic storm. Useful distinguishing features include age group (pediatric, young adult, adult and all), presence or absence of triggering factors (number of “+” sign correlates with stronger relationship; “+/−” represents inconsistent correlation; “−” represents no correlation), time course, movement disorder phenomenology, associated neurological signs, altered mental status and autonomic instability

Abbreviations: *P* pediatric, *YA* young adults, *A* adults, *All* all age groups



**Additional file 1:** Video segment 1. This video segment demonstrates the challenge of diagnosing dystonic storm clinically. *Patient 1* demonstrates parkinsonism-hyperpyrexia syndrome in a Parkinson’s disease patient (neuroleptic malignant syndrome) resulting from discontinuation of dopaminergic medications. Within 24 h, he developed worsening encephalopathy, severe generalized rigidity especially in the lower extremities, myoclonic jerks, and hyperthermia. Laboratory findings revealed eleveated creatine kinase (CK) of over 1,950 IU and leukocytosis. He was admitted and treated in the intensive care unit. *Patient 2* is a young man who developed serotonin syndrome soon after initiation of a selective serotonin reuptake inhibitor for depression. The video demonstrates almost constant prominent low amplitude myoclonic jerks of both legs. Also not shown here were encephalopathy, mild hyperthermia and mild eleveated serum CK. *Patient 3* is a young woman with anti-N-methyl-D-aspartate receptor (anti-NMDAR) encephalitis. She had mild dyskinesias in the perioral region and left cheek. She appeared to be awake but did not respond to questions or commands. Her right hand showed posturing with low amplitude stereotypic movements. She was found to have an ovarian teratoma, and improved markedly after receiving immunotherapies (not shown). (MP4 92862 kb)


## Mechanism of dystonic storm

**Fig. 1:**
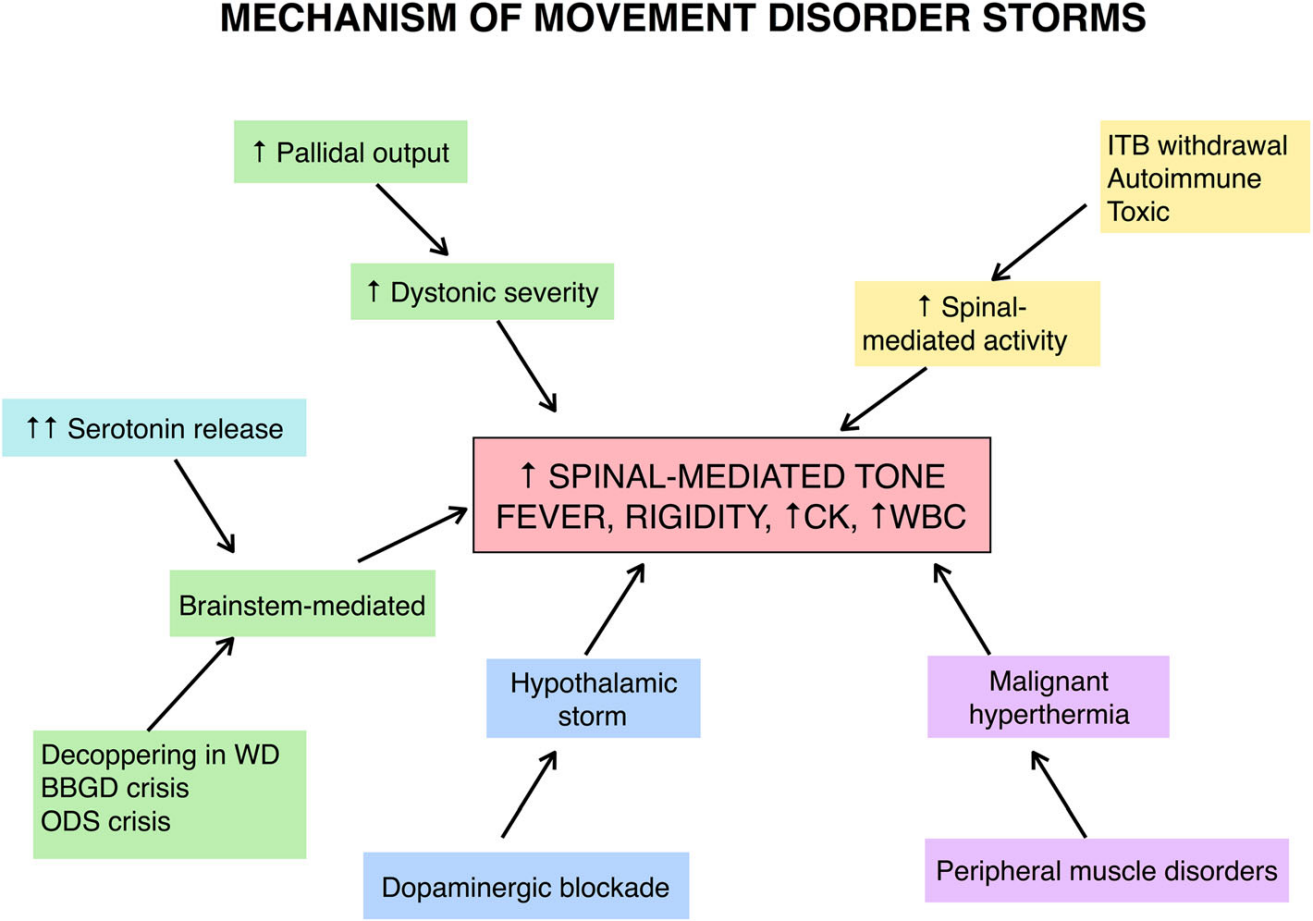
Pathophysiology of movement disorder emergencies (“storms”) including dystonic storm. This figure demonstrates proposed the pathophysiology of several movement disorder emergencies including dystonic storm (*green*), serotonin syndrome (increased serotonin leading to brainstem-mediated process; *turquoise*), malignant hyperthermia (*purple*) from peripheral muscle disorders such as secondary to ryanodine receptor (*RYR1*) mutations, neuroleptic malignant syndrome (from dopaminergic blockade leading to hypothalamic storm; *blue*), and others which lead to increased spinal-mediated rigidity (such as intrathecal baclofen withdrawal, autoimmune disesase e.g. stiff person syndrome or progressive encephalomyelitis with rigidity and myoclonus, and toxin-mediated disorders e.g. tetanus; *yellow*). These disorders lead to the final common pathway characterized by increased spinal-mediated muscle tone, fever, rigidity, elevated creatine kinase and leukocytosis. Abbreviations: BBGD, biotin-thiamine-responsive basal ganglia disease; CK, creatine kinase; ITB, intrathecal baclofen; ODS, osmotic demyelinating syndrome; WBC, *white* blood cells; WD, Wilson’s disease

The mechanism and neurotransmitter systems involved in dystonic storm remain unknown. We present a hypothetical framework for conceptualizing possible mechanisms of various storm conditions in Fig. 1. In patients with underlying dystonia, increased dystonia severity may result from increased pallidal output. Intrathecal baclofen (ITB) withdrawal, autoimmune disorders such as stiff person syndrome or progressive encephalomyelitis with rigidity and myoclonus, and toxic etiologies (such as tetanus) may lead to spinal-mediated overactivity. Decoppering in Wilson’s disease (especially with D-penicillamine), crisis in biotin-thiamine-responsive basal ganglia disease, acute osmotic demyelinating syndrome and serotonin syndrome may be mediated through the brainstem. Neuroleptic malignant syndrome from dopaminergic blockade may lead to hypothalamic storm. The mechanism of malignant hyperthermia likely originates peripherally, as evidenced by associated muscle disorders such as myopathies secondary to ryanodine receptor (*RYR1*) mutations. Neuroleptic malignant syndrome, serotonin syndrome and malignant hyperthermia may share a final common pathway, characterized by an increase in spinal-mediated tone and associated signs and symptoms including fever, hyperCKemia and leukocytosis. Various neurotransmitter systems including dopaminergic, serotoninergic, GABAergic, glycinergic and glutamatergic systems are likely involved in these disorders.

## Dystonic storm: clinical features

Dystonic storm usually occurs in patients who are already known to have dystonia, and are usually followed in movement disorders clinic. De novo presentation of a patient in dystonic storm is rare. Mean prior dystonia duration is 6 years, with an average age at presentation of 14 years [6]. Dystonic storm tends to occur in patients with severe or poorly controlled dystonia at baseline [2, 9], and one-third of events are unprovoked [6, 10]. Among provoked events, triggers include infection (50%) and medication change (30%). Examples of the latter include ITB withdrawal [11, 12], reduction or withdrawal of other dystonia medications or initiation of D-penicillamine in Wilson’s disease [13, 14]. Other examples include introduction of clonazepam (paradoxically it can also be used as treatment of dystonic storm) [1], initiation of zinc in Wilson’s disease [15] or zolpidem discontinuation in Parkinson’s disease [16]. Other provoking factors include surgical procedures and deep brain stimulation (DBS) failure [17, 18]. As DBS has become more widespread, there have been more reports of dystonic storm from DBS failure, especially due to battery issues or at the end of battery life [19].

**Fig. 2:**
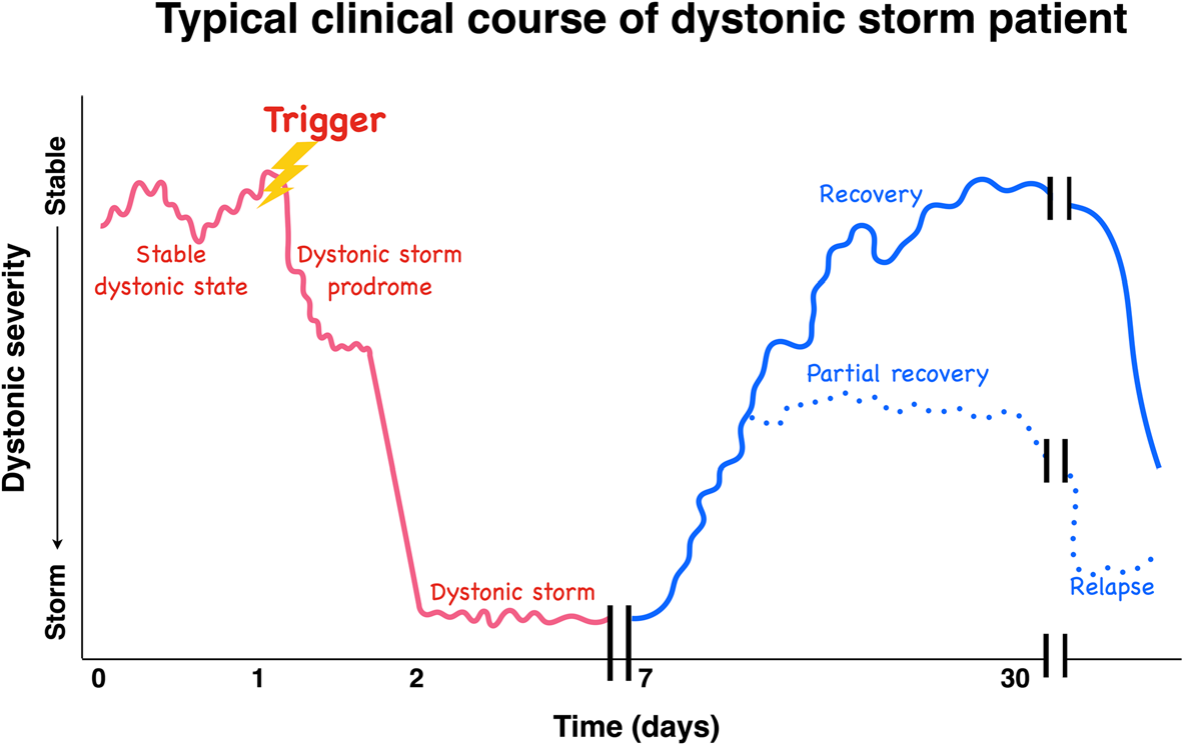
Typical clinical course of dystonic storm. This figure demonstrates the typical clinical course of dystonic storm. Dystonic storm typically occurs in patients with known underlying dystonia seen on the left. Two-thirds of events are provoked by triggers such as infection or medication changes. Dystonia then worsens slightly from baseline, called the “dystonic storm prodrome”. If not detected or treated properly, dystonia rapidly escalates to “dystonic storm”, which usually lasts days to 2–4 weeks. With appropriate dystonia-specific and supportive therapies, most patients will gradually recover back to baseline (full recovery) or with residual deficits (partial recovery). Relapses are not uncommon



In some patients a prodrome of dystonic storm may occur, where dystonia is worsened from baseline but not as severe as in true storm (Fig. 2 and Additional file 2: Video segment 2). Once true storm begins, it usually lasts 2–4 weeks with gradual recovery. However even with treatment, mortality remains 10% [6]. If the patient survives, they may return back to their baseline severity of dystonia (full recovery) or be left with some residual deficits (partial recovery) [6]. Relapses are common, and patients with a history of one event should be monitored for recurrence.

**Additional file 2:** Video segment 2. This video demonstrates the clinical course of dystonic storm. The first segment shows the prodromal phase of dystonic storm (Fig. 1) in a young boy with DYT1 generalized dystonia. During this phase, dystonia severity was worse from his baseline. The latter part of the video demonstrates a patient in true dystonic storm phase (the trough portion of the graph in Fig. 1). He had severe dystonic posturing of bilateral arms and legs, seen as flexion of shoulders, elbows, hips and knees, giving a “pretzel-like” feature. (MP4 61017 kb)


Typical clinical scenarios for dystonic storm include DYT1 dystonia [20, 21], Wilson’s disease (typically after initiation of D-penicillamine) [13, 14], and cerebral palsy (CP) with infectious trigger [1, 6, 22]. Other scenarios for dystonic storm are listed in Table 2.

**Table 2:** List of scenarios for dystonic storm

Primary dystonia	
	**▪** DYT1 generalized dystonia [20, 21, 26]
	**▪** Primary generalized DYT1-negative dystonia [1]
Structural brain injury	
	**▪** Cerebral palsy [15, 22]
	**▪** Traumatic brain injury [12]
Metabolic disorders affecting subcortical areas	
	**▪** Mitochondrial disorders including Leigh’s syndrome [6]
	**▪** Glutaric aciduria type I [44]
	**▪** Wilson’s disease [13–15]
Progressive heredodegenerative dystonia	
	**▪** Pantothenate kinase-associated neurodegeneration [15, 31, 34, 40, 45, 46]
	**▪** Neuroacanthocytosis [1]
	**▪** Neuronal ceroid lipofuscinosis (Batten’s disease) [41]
Miscellaneous	
	**▪** Genetic syndromes e.g. ARX syndrome [47], megalencephalic leukoencephalopathy with subcortical cysts (MLC) [10], SOX-2 anophthalmia syndrome [48]
	**▪** Tardive dystonia [30, 35]
	**▪** Post-traumatic dystonia [1]
	**▪** Ataxia-telangiectasia [49]
	**▪** Familial idiopathic hypoparathyroidism [50]

This table demonstrates scenarios where dystonic storm emerges from. Of note, the scenarios represent dystonic disorders at baseline, rather than triggering factors (e.g. withdrawal from medications such as baclofen or deep brain stimulation battery failure) which are not included here

Although there is currently no rigid border or strict clinical criteria to diagnose dystonic storm, it is crucial for clinicians to be vigilant when encountering patients with worsening dystonia from their baseline in order to be able to identify dystonic storm early in prodromal phase and promptly initiate interventions. Given no clear border when patients are progressing to dystonic storm, clinical judgment is required in order to find balance of early identification but not over-treating. However under-treatment is likely to be more harmful. Some dystonic disorders can have acute presentation, and it is important to recognize in order not to misidentify them as dystonic storm. Table 3 and Additional file 3: Video segment 3 demonstrates examples of metabolic and genetic disorders that can present with acute dystonia for reference.

**Table 3:** Metabolic and genetic disorders that can present with acute dystonia

**▪** Wilson’s disease	
**▪** Rapid-onset dystonia parkinsonism (RDP)	
**▪** Biotin-thiamine-responsive basal ganglia disease	
**▪** Mitochondrial encephalopathy	
**▪** Osmotic demyelinating syndrome including central pontine and extra-pontine myelinolysis	

This table demonstrates a list of metabolic and genetic disorders that can present with acute dystonia



**Additional file 3:** Video segment 3. This segment demonstrates examples of movement disorders that can present with acute dystonia. *Patients 1 and 2* have Wilson’s disease. Patient 1 is a teenage girl with a prominent risor grin and difficutly performing left finger tapping due to prominent dystonia. Spastic dysarthria was present. When walking, marked, left greater than right dystonic posturing of both arms was seen. Kayser-Fleischer rings are seen in Patient 2 as brown pigmentation around the entire circumference of the corneal limbi. *Patient 3* is a girl with an intermediate phenotype between alternating hemiplegia of childhood and rapid-onset dystonia parkinsonism (DYT12 dystonia) with a genetically confirmed mutation in the *ATP1A3* gene. This home video demonstrates an episode of head tilt and turn with oculogyria lasting over 60 s (this video did not capture the entire episode). During the event, she cried and was uncomfortable, but consciousness remained intact. She was treated with levodopa 300 mg/day with resolution of dystonic episodes. *Patient 4* had acute dystonia and parkinsonism from osmotic demyelinating syndrome after rapid correction of hyponatremia. Both pontine and extrapontine myelinolysis (bilateral putamen) were present. He had marked facial dystonia, a risor grin and prominent lingual dystonia (not shown) which led to inability to speak. (MP4 109557 kb)


## Management decisions in dystonic storm

**Fig. 3:**
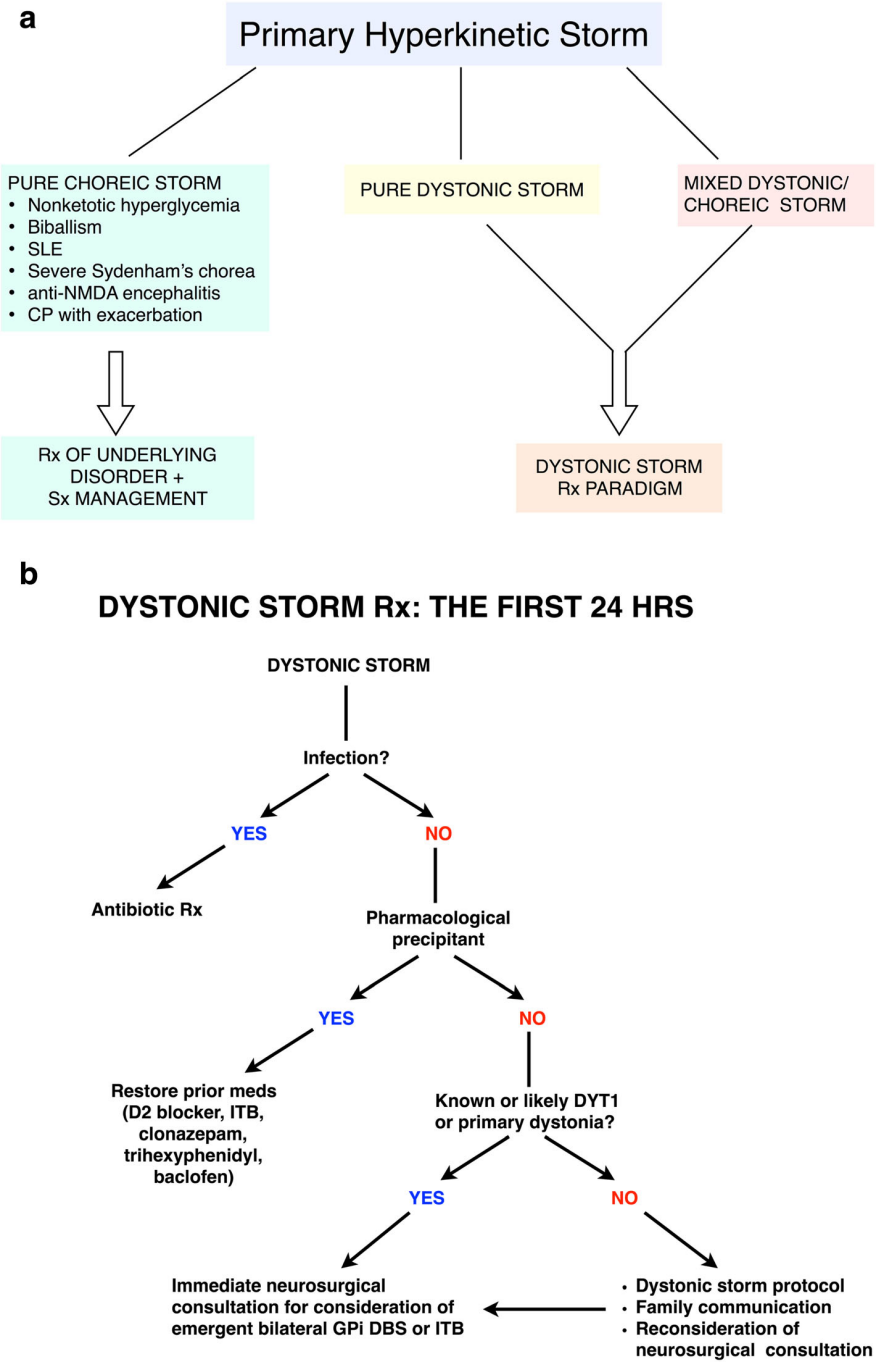
Thought process in the first 24 h. **a** demonstrates an algorithm for management in the first 24 h. After excluding other movement disorders emergencies outlined in Table 1, dystonic or mixed dystonic/choreic storms should be distinguished from pure choreic storm. **b** demonstrates a flow chart of the subsequent thought process in the first 24 h. The goals are to 1) identify triggers such as infection and medication changes, and treat accordingly, and 2) address if the patient is a candidate for GPi DBS or ITB pump. Abbreviations: D2, D2 dopamine receptor; GPi DBS, globus pallidus interna deep brain stimulation; hrs, hours; ITB, intrathecal baclofen; Rx, treatment

Once dystonic storm has begun, management must be initiated promptly. Hospital admission is generally required and patients should be admitted to the intensive care unit. The strategy of management differs in the acute and subacute periods. Here we divide the management into the first 24 h (Fig. 3a and b) vs. the next 2–4 weeks (Table 4). This distinction reflects the critical nature of the decisions that must be made within the first 24 h in order to minimize morbidity and mortality.

**Table 4:** Dystonia-specific and supportive therapies: thought process for the next 2–4 weeks

DYSTONIA SPECIFIC Rx^a^	SUPPORTIVE Rx
Anticholinergics	1st line: IV midazolam
DA receptor blockers^b^	2nd line: Propofol
TBZ	3rd line: Non-depolarizing neuromuscular blockers or Barbiturates
Clonidine	
Baclofen + Assorted drugs	

^a^Combination is typically required

^b^Should be avoided when dystonic storm developing from underlying tardive dystonia

This table demonstrates the list of dystonia-specific and supportive therapies. Dystonia-specific medications alone are often ineffective in isolation to control dystonic storm. Combinations of drugs are generally required. Intravenous midazolam is typically employed as a first-line agent, whereas propofol is second-line. In refractory cases, third-line agents include non-depolarizing neuromuscular blocking agents (such as pancuronium) or barbiturates

Abbreviations: *DA* dopamine, *IV* intravenous, *Rx* treatment, *TBZ* tetrabenazine

The main aims of management during the first 24 h are 1) to identify triggers and their specific treatments and 2) to assess whether or not the patient is a candidate for globus pallidus interna (GPi) DBS or ITB therapy. Some may argue that invasive therapies such as GPi DBS or ITB should be considered as last resort treatments, to be used when dystonic storm is refractory to other therapies [23]. Nevertheless, with increased utilization of DBS in the past decade, the pendulum may be shifting away from this approach. An increasing number of reports have demonstrated excellent outcomes of GPi DBS and ITB in dystonic storm [10, 21, 24–37], especially in DYT1 generalized dystonia and other primary dystonia cases (Additional file 4: Video segment 4). Due to the dramatic impact on patient outcome, the modern trend is to advocate a tiered approach (based on diagnosis e.g. generalized DYT1 dystonia and severity) [5, 38] with consideration of GPi DBS or ITB therapy [21, 26–29, 39]. Patients in dystonic storm often improve immediately with DBS [8, 24], as opposed to the delayed benefit (weeks to months) of DBS in other forms of dystonia. ITB use may be limited by its complications, especially hardware failure and infection [37]. Pallidotomy [31, 40–42] is rarely used unless DBS is unavailable.



**Additional file 4:** Video segment 4. This video demonstrates two patients who benefitted from GPi DBS. *Patient 1* (Video courtesy of Catherine Cho, MD) is a young boy with DYT1 generalized dystonia. He presented with marked and rapid worsening dystonia from his baseline. Prominent truncal and neck extension, bilateral hip extension and knee flexion, along with severe pain were present. He underwent bilateral GPi DBS within 24 h after onset with immediate improvement in dystonia, espeically in his trunk and legs. However, dystonia of both arms with intermittent involuntary flexion of bilateral elbows remained quite prominent. He continued to improve further after several sessions of DBS programming over 1–2 years. *Patient 2* is a boy with severe generalized dystonia involving trunk, bilateral arms and legs who underwent bilateral GPi DBS. He was unable to stand, and his trunk showed marked lateral flexion. When the implantable pulse generator became infected, he developed marked worsening of dystonia from his baseline with severe truncal extension and posturing of both arms and legs. His dystonia was so severe that he had to be on the floor. Re-establishment of the DBS system led to improvement of dystonia over the next several months. On follow-up visit, he had residual mild-to-moderate dystonia of the left arm and the left foot, seen as elbow flexion and foot inversion, but was able to walk independently. (MP4 108739 kb)


Another important question when approaching a critically ill hyperkinetic patient is to distinguish dystonic and choreic storm from other movement disorder emergencies, as delineated in Table 1 (Additional file 5: Video segment 5). Predisposing conditions in both pure dystonic storm and mixed dystonic/choreic storm are often similar, for example in the clinical scenarios of CP, traumatic brain injury and Leigh’s syndrome. Patients with dystonic storm may also have co-existing chorea. While dystonic and choreic storms appear similar, the management of choreic storm should focus on identifying and treating the underlying trigger. Nonketotic hyperglycemia, biballism, lupus, severe Sydenham’s chorea, anti-N-methyl-D-aspartate receptor (anti-NMDAR) encephalitis and CP with exacerbation can cause choreic storm. The treatment of dystonic and choreic storms overlaps somewhat: neuroleptics, tetrabenazine and GPi DBS [24], with the exception of anticholinergics (which can worsen chorea). Nevertheless, the therapeutic strategy for pure dystonic or mixed dystonic/choreic storm should follow the dystonic storm paradigm discussed below (Fig. 3).



**Additional file 5:** Video segment 5. This video segment demonstrates pure choreic storm (*Patients 1–3*). *Patient 1* had non-ketotic hyperglycemia. He presented with prominent chorea in the orobuccolingual region and bilateral arms. *Patient 2* also had non-ketotic hyperglycemia. Nevertheless, chorea in this patient mainly affected the left arm and leg, and to a lesser extent, the right leg without orobuccolingual chorea. *Patient 3* is a young boy with Sydenham’s chorea. He had history of streptococcal pharyngitis a few months prior. Prominent chorea involved bilateral arms, trunk and, to a lesser extent, perioral region. (MP4 64087 kb)


A proposed flow chart for dystonic storm management in the first 24 h is demonstrated in Fig. 3b. Triggers such as infection or medication change should be identified and treated accordingly, for example by initiating antibiotics or restoring prior medications such as dopamine receptor blockers, ITB, clonazepam or trihexyphenidyl. If the patient is known or likely to have DYT1 generalized dystonia, immediate neurosurgical consultation should be obtained to consider emergent bilateral GPi DBS. We would also suggest this approach for patients with DYT1-negative primary dystonia.

If the patient does not meet these criteria, effective communication with the patient’s family is essential. The efficacy of medical management of dystonic storm is only 10% [6] and mortality remains about 10% [6]. Primary questions to be answered are 1) What are the family’s goals of care? and 2) Is the patient a neurosurgical candidate for ITB or GPi DBS (or ablative surgery) should medical therapies fail? If the patient is not a surgical candidate, management remains medical. Table 4 demonstrates the list of usual medical treatments employed over the next 2–4 weeks. These can be separated into dystonia specific therapies and supportive treatment. Dystonia specific treatment includes anticholinergics, dopamine receptor blockers, tetrabenazine, clonidine, baclofen and assorted drugs. The so-called “Marsden cocktail”, a combination of tetrabenazine 75 mg/day, pimozide and benzhexol, was first employed in England [3]. Management remains empiric, without evidence-based guideline. Regardless of the combinations, the key is to use multiple high-dose anti-dystonic agents to control or abort storm, rather than slowly titrating one agent at a time. Of note, dopamine receptor blockers should be avoided in dystonic storm developing from underlying tardive dystonia.

Along with dystonia-specific treatment, supportive measures include airway protection, sedation and pain control. Dystonia-specific therapies alone are often ineffective, or may require days to weeks before achieving improvement. Anesthetic agents typically used in dystonic storm include intravenous midazolam, propofol, and barbiturates. Intravenous midazolam is generally selected as a first choice due to its muscle relaxation effect, short half-life, rapid onset of action, and good cardiovascular profile [1, 9, 43]. If the symptoms are not controlled, propofol, an anesthetic with relatively short half-life, is generally considered as a second line agent. Third line drugs are non-depolarizing paralytic agents such as pancuronium and barbiturates. Of note, depolarizing neuromuscular blocking agents such as suxamethonium should be avoided due to their potential risk of rhabdomyolysis.

## Conclusions and Future directions

Dystonic storm is rare and evidence-based guidelines for management are lacking. The true incidence and prevalence of dystonic storm remains unclear. Evidence-based guidelines addressing medication selection, drug order, timing, dose, polypharmacy, the role of intravenous sedation and intubation, as well as the timing and patient selection criteria for DBS and ITB are desperately needed. In addition, early consideration of DBS remains a challenging issue in some countries where this treatment modality is not available.

## Abbreviations

anti-NMDAR: Anti-N-methyl-D-aspartate receptor
CK: Creatine kinase
CP: Cerebral palsy
DBS: Deep brain stimulation
GPi: Globus pallidus interna
ITB: Intrathecal baclofen
